# A mutation update on the LDS‐associated genes *TGFB2/3* and *SMAD2/3*

**DOI:** 10.1002/humu.23407

**Published:** 2018-03-06

**Authors:** Dorien Schepers, Giada Tortora, Hiroko Morisaki, Gretchen MacCarrick, Mark Lindsay, David Liang, Sarju G. Mehta, Jennifer Hague, Judith Verhagen, Ingrid van de Laar, Marja Wessels, Yvonne Detisch, Mieke van Haelst, Annette Baas, Klaske Lichtenbelt, Kees Braun, Denise van der Linde, Jolien Roos‐Hesselink, George McGillivray, Josephina Meester, Isabelle Maystadt, Paul Coucke, Elie El‐Khoury, Sandhya Parkash, Birgitte Diness, Lotte Risom, Ingrid Scurr, Yvonne Hilhorst‐Hofstee, Takayuki Morisaki, Julie Richer, Julie Désir, Marlies Kempers, Andrea L. Rideout, Gabrielle Horne, Chris Bennett, Elisa Rahikkala, Geert Vandeweyer, Maaike Alaerts, Aline Verstraeten, Hal Dietz, Lut Van Laer, Bart Loeys

**Affiliations:** ^1^ Center of Medical Genetics University of Antwerp and Antwerp University Hospital Antwerp Belgium; ^2^ Medical Genetics Unit Department of Medical and Surgical Sciences University of Bologna, Policlinico Sant'Orsola‐Malpighi Bologna Italy; ^3^ Department of Molecular and Clinical Sciences Marche Polytechnic University Ancona Italy; ^4^ Department of Bioscience and Genetics National Cerebral and Cardiovascular Center Research Institute Suita Osaka Japan; ^5^ Department of Molecular Pathophysiology Osaka University Graduate School of Pharmaceutical Sciences Suita Osaka Japan; ^6^ Department of Medical Genetics Sakakibara Heart Institute Tokyo Japan; ^7^ McKusick‐Nathans Institute of Genetic Medicine Johns Hopkins University School of Medicine Baltimore Maryland; ^8^ Thoracic Aortic Center, Departments of Medicine and Pediatrics Massachusetts General Hospital Harvard Medical School Boston; ^9^ Cardiovascular Medicine Stanford University Medical Center Stanford California; ^10^ East Anglian Regional Genetics Service Cambridge University Hospitals NHS Foundation Trust Addenbrooke's Hospital Cambridge UK; ^11^ Department of Clinical Genetics Erasmus University Medical Center Rotterdam The Netherlands; ^12^ Department of Clinical Genetics Maastricht University Medical Center Maastricht The Netherlands; ^13^ Department of Medical Genetics University Medical Center Utrecht Utrecht The Netherlands; ^14^ Department of Clinical Genetics Academic Medical Center Amsterdam The Netherlands; ^15^ Department of Child Neurology Brain Center Rudolf Magnus University Medical Center Utrecht Utrecht The Netherlands; ^16^ Department of Cardiology Erasmus Medical Center Rotterdam The Netherlands; ^17^ Victorian Clinical Genetics Services Murdoch Children's Research Institute Melbourne Australia; ^18^ Centre de Génétique Humaine Institut de Pathologie et de Génétique (IPG) Gosselies (Charleroi) Belgium; ^19^ Center for Medical Genetics Ghent University Hospital and Ghent University Ghent Belgium; ^20^ Department of Diagnostic Cardiology Clinique St Luc Bouge (Namur) Belgium; ^21^ Department of Pediatrics Maritime Medical Genetics Service IWK Health Centre Dalhousie University Halifax Nova Scotia Canada; ^22^ Department of Clinical Genetics, Rigshospitalet Copenhagen University Hospital Copenhagen Denmark; ^23^ Department of Clinical Genetics St. Michael's Hospital Bristol UK; ^24^ Department of Clinical Genetics Leiden University Medical Center Leiden The Netherlands; ^25^ Department of Medical Genetics, Children's Hospital of Eastern Ontario Children's Hospital of Eastern Ontario Research Institute Ottawa Ontario Canada; ^26^ Centre de Génétique Humaine, Hôpital Erasme Université Libre de Bruxelles Belgium; ^27^ Department of Human Genetics Radboud University Nijmegen Medical Center Nijmegen The Netherlands; ^28^ Maritime Medical Genetics Service IWK Health Centre Halifax Nova Scotia Canada; ^29^ Department of Medicine (Cardiology) and School of Biomedical Engineering Dalhousie University Halifax Nova Scotia Canada; ^30^ Department of Clinical Genetics Chapel Allerton Hospital Leeds Teaching Hospitals NHS Trust Leeds UK; ^31^ Department of Clinical Genetics Oulu University Hospital, University of Oulu Oulu Finland

**Keywords:** aneurysm, Loeys–Dietz syndrome, *SMAD2*, *SMAD3*, *TGFB2*, *TGFB3*

## Abstract

The Loeys–Dietz syndrome (LDS) is a connective tissue disorder affecting the cardiovascular, skeletal, and ocular system. Most typically, LDS patients present with aortic aneurysms and arterial tortuosity, hypertelorism, and bifid/broad uvula or cleft palate. Initially, mutations in transforming growth factor‐β (TGF‐β) receptors (*TGFBR1* and *TGFBR2*) were described to cause LDS, hereby leading to impaired TGF‐β signaling. More recently, TGF‐β ligands, *TGFB2* and *TGFB3*, as well as intracellular downstream effectors of the TGF‐β pathway, *SMAD2* and *SMAD3*, were shown to be involved in LDS. This emphasizes the role of disturbed TGF‐β signaling in LDS pathogenesis. Since most literature so far has focused on *TGFBR1/2*, we provide a comprehensive review on the known and some novel *TGFB2/3* and *SMAD2/3* mutations. For *TGFB2* and *SMAD3*, the clinical manifestations, both of the patients previously described in the literature and our newly reported patients, are summarized in detail. This clearly indicates that LDS concerns a disorder with a broad phenotypical spectrum that is still emerging as more patients will be identified. All mutations described here are present in the corresponding Leiden Open Variant Database.

## 1 BACKGROUND

The Loeys–Dietz syndrome (LDS, MIM# 609192, 610168, 613795, 614816, 615582) is an autosomal dominant connective tissue disorder with widespread systemic involvement. In 2005, Loeys and Dietz were the first to describe this disorder, which is—in its most typical presentation—characterized by vascular tortuosity and aneurysm in association with craniofacial and skeletal manifestations (Loeys et al., 2005; Loeys et al., 2006). On the one hand, LDS shows significant clinical overlap with Marfan syndrome (MFS, MIM# 154700), with vascular and skeletal features including aortic root aneurysm, arachnodactyly, scoliosis, and pectus deformity. On the other hand, a clear distinction between LDS and MFS can be made based on typical LDS findings such as widespread aortic/arterial aneurysm and tortuosity, club foot, craniosynostosis, hypertelorism, and bifid/broad uvula or cleft palate. Other LDS‐specific features can include cervical spine malformation and/or instability, translucent skin with easy bruising and dystrophic scars, severe allergic tendency, and bowel inflammation including eosinophilic esophagitis/gastritis and/or inflammatory bowel disease. Soon after the initial description, the phenotypic spectrum was expanded to less syndromic presentations, including those that overlap with vascular Ehlers–Danlos syndrome (Loeys et al., 2005; Loeys et al., 2006). Mutations in the genes encoding the transforming growth factor β (TGF‐β) receptor I (*TGFBR1*) and TGF‐β receptor II (*TGFBR2*) subunits were the first reported genetic causes of LDS. More recently, mutations in four additional genes, namely the mothers against decapentaplegic homolog 2 and 3 (*SMAD2/3*) and the TGF‐β 2 and 3 ligand (*TGFB2/3*), have been shown to cause an LDS‐like phenotype (Bertoli‐Avella et al., 2015; Boileau et al., 2012; Lindsay et al., 2012; Micha et al., 2015; van de Laar et al., 2011).

*SMAD2* and *SMAD3*, located on the long arm of chromosome 18 and 15, span about 130 kb and consist of 11 and 9 exons, respectively. Both the SMAD2 and SMAD3 proteins belong to the receptor‐activated (R)‐SMAD family, intracellular effectors of the canonical TGF‐β signaling pathway. The activating ligands of this pathway include TGF‐β2 and TGF‐β3, which are encoded by the *TGFB2* and *TGFB3* genes. These genes include 8 and 7 exons and are positioned on the long arm of chromosome 1 and 14, respectively. Including *TGFBR1/*2, all genes related to LDS spectrum disorders are part of the TGF‐β signaling pathway. Additionally, fibrillin‐1, which is encoded by the *FBN1* gene and deficient in MFS, binds to the large latent TGF‐β complex and contributes to TGF‐β bioavailability and activation (Chaudhry et al., 2007; Isogai et al., 2003; Neptune et al., 2003). This clearly highlights the key role of the TGF‐β pathway in the pathogenesis of aortic aneurysm development in LDS and LDS‐like disorders. However, the exact pathogenic mechanisms remain controversial.

Initially, it was suggested that two types of LDS could be distinguished: patients with LDS type 1 (LDSI) displayed typical craniofacial features, where LDS type 2 (LDSII) patients had more pronounced cutaneous features. Recently, this classification was revised and these LDS types are now thought to be part of a phenotypic spectrum of disease. Therefore, LDS type 1 (LDS1) and LDS type 2 (LDS2) now designate to the disease‐responsible genes; *TGFBR1* and *TGFBR2*, respectively. Mutations in *SMAD3* were initially described as the genetic cause of aneurysms‐osteoarthritis syndrome, but because of the many overlapping clinical features with LDS, including hypertelorism, bifid uvula, arterial tortuosity, and widespread and aggressive aneurysms, it is now also classified as LDS type 3 (LDS3) (MacCarrick et al., 2014). Similarly, patients with mutations in *TGFB2* share clinical manifestations with LDS, and are for this reason now diagnosed with LDS type 4 (LDS4). More recently, patients with mutations in *SMAD2* and *TGFB3* (LDS5) were also shown to present with LDS‐like features (Bertoli‐Avella et al., 2015; Micha et al., 2015). It is thus suggested that a mutation in any of these six genes in combination with the presence of arterial aneurysm or dissection should be sufficient for the diagnosis of LDS (MacCarrick et al., 2014). Since most literature so far has focused on LDS1 and LDS2, this review will highlight known and novel *SMAD2/3* and *TGFB2/3* mutations and will summarize and compare the clinical features of affected individuals with those reported in the literature.

## 2 VARIANTS

Each published mutation was checked for accuracy and compared to the respective wild‐type reference sequence. When a different reference sequence was used, nucleotide and codon numbers were converted so their annotation matched with reference transcripts: NM_001135599.2 for *TGFB2*, NM_003239.3 for *TGFB3*, NM_005901.5 for *SMAD2* and NM_005902.3 for *SMAD3*. The A nucleotide of the translation initiation codon (ATG) was designated as position +1. All variants are submitted to the corresponding Leiden Open Variant Database (LOVD) (Fokkema et al., 2011) http://143.169.238.105/LOVD/genes/TGFB2; http://143.169.238.105/LOVD/genes/TGFB3; http://143.169.238.105/LOVD/genes/SMAD2; http://143.169.238.105/LOVD/genes/SMAD3).

**Figure 1:**
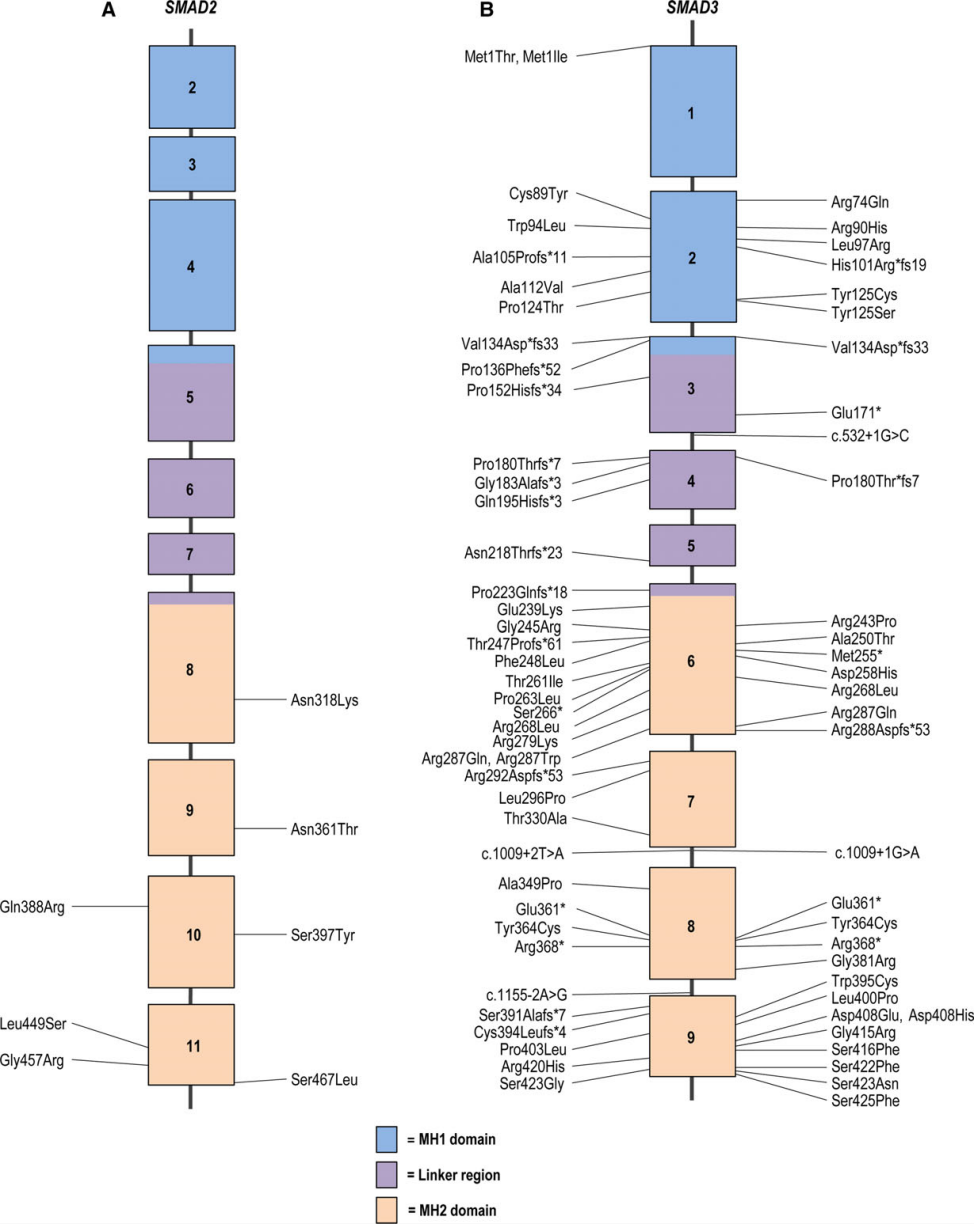
Schematic representation of the *SMAD2* and *SMAD3* gene with their protein coding domains. Boxes represent exons 1–11 and 1–9, respectively. On the left side of the schematic are the previously reported mutations, whereas on the right side mutations identified in this study are described. For *SMAD2*, the first depicted exon is exon 2 because exon 1 is 5′UTR. Mutations are annotated at the protein level, with exception of splice site mutations (reference transcript: NM_005901.5 and NM_005902.3 for *SMAD3*)

**Figure 2:**
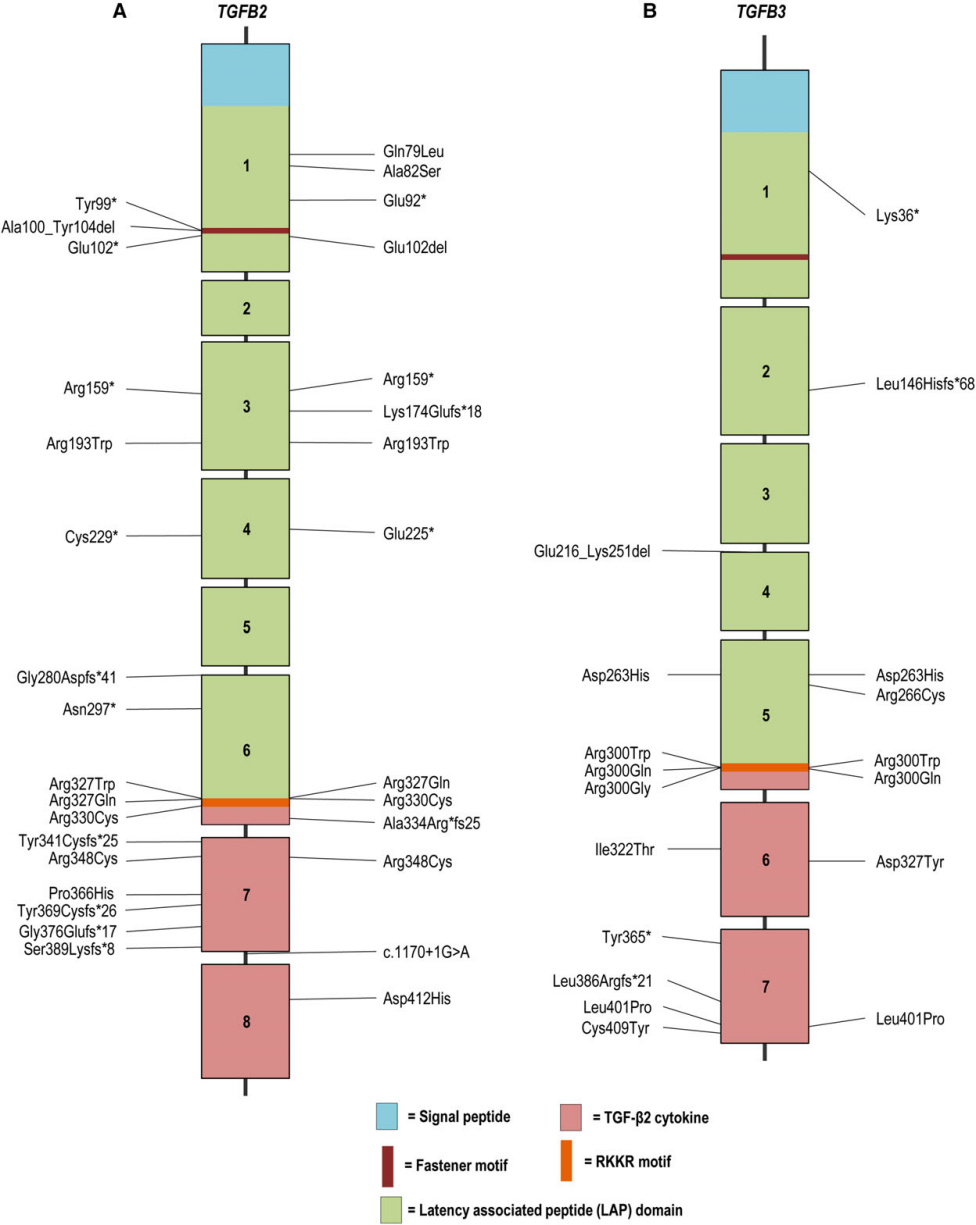
Schematic representation of the *TGFB2* and *TGFB3* gene with their protein coding domains. Boxes represent exons 1–8 and 1–7, respectively. On the left side of the schematic are the previously reported mutations, whereas on the right side mutations identified in this study are described. Mutations are annotated at the protein level (reference transcript: NM_001135599.2 for *TGFB2* and NM_003239.3 for *TGFB3*)

We report 7, 67, 30, and 15 different variants for *SMAD2*, *SMAD3*, *TGFB2*, and *TGFB3*, respectively (Tables 1, 2, and 3 and Figures 1 and 2). These variants were either published as full article cited in PubMed or newly identified in this study. We did not include variants from abstracts (without full article) or the ClinVar database since little or no clinical information is available on these individuals. Except for the five variants listed in Table 4, these mutations are not found in the ExAC database (http://exac.broadinstitute.org/; accessed April 2017). To obtain an indication on the pathogenicity, all variants are classified according to the ACMG guidelines (Suppl. Table S1) (Richards et al., 2015).

**Table 1:** Previously described and novel *SMAD3* gene mutations

Exon	c‐Notation	p‐Notation	Domain	Reference	Times reported	Effect
1	c.2T > C	p.Met1Thr	MH1	Haller et al. (2015)	1	Pathogenic
1	c.3G > A	p.Met1Ile	MH1	Fitzgerald et al. (2014)	1	Pathogenic
**2**	**c.221G > A**	**p.Arg74Gln**	**MH1**	**Current study**	**2**	**Likely pathogenic**
2	c.266G > A	p.Cys89Tyr	MH1	Zhang et al. (2015)	1	Pathogenic
**2**	**c.269G > A**	**p.Arg90His**	**MH1**	**Current study**	**1**	**Likely pathogenic**
2	c.281G > T	p.Trp94Leu	MH1	Blinc et al. (2015)	1	VUS
**2**	**c.290T > G**	**p.Leu97Arg**	**MH1**	**Current study**	**1**	**VUS**
**2**	**c.300_301insAGGGCCGGCAGGC**	p.His101Argfs*14	**MH1**	**Current study**	**1**	**Pathogenic**
2	c.313delG	p.Ala105Profs*11	MH1	van de Laar et al. (2012)	1	Pathogenic
2	c.335C > T	p.Ala112Val	MH1	(Regalado et al., 2011)	1	Likely pathogenic
2	c.370C > A	p.Pro124Thr	MH1	(Garcia‐Bermudez et al., 2017)	1	VUS
**2**	**c.374A > G**	**p.Tyr125Cys**	**MH1**	**Current study**	**1**	**Likely pathogenic**
**2**	**c. 374A > C**	**p.Tyr125Ser**	**MH1**	**Current study**	**1**	**Likely pathogenic**
**3**	**c.401‐6G > A**	p.Val134Aspfs*33	**MH1**	**(** **Campens et al.,** 2015 **), Current study**	**2**	**Pathogenic**
3	c.401_405dup	p.Pro136Phefs*52	MH1	(Berthet et al., 2015)	1	Pathogenic
**3**	**c.455delC**	p.Pro152Hisfs*34	**Linker**	**(** **Courtois et al.,** 2017 **)**	**1**	**Pathogenic**
**3**	**c.511G > T**	p.Glu171*	**Linker**	**Current study**	**1**	**Pathogenic**
**3**	**c.532+1G > C**		**Linker**	**Current study**	**1**	**Pathogenic**
4	c.539_540insC	p.Pro180Thrfs*7	Linker	(van de Laar et al., 2012), Current study	2	Pathogenic
4	c.546delT	p.Gly183Alafs*3	Linker	(Campens et al., 2015)	1	Pathogenic
4	c.584_585insTC	p.Gln195Hisfs*3	Linker	(Campens et al., 2015)	1	Pathogenic
5	c.652delA	p.Asn218Thrfs*23	Linker	(Regalado et al., 2011)	1	Pathogenic
6	c.668delC	p.Pro223Glnfs*18	Linker	(Aubart et al., 2014)	1	Pathogenic
6	c.715G > A	p.Glu239Lys	MH2	(Regalado et al., 2011), (Campens et al., 2015)	4	Pathogeic
**6**	**c.728G > C**	**p.Arg243Pro**	**MH2**	**Current study**	**1**	**Likely pathogenic**
6	c.733G > A	p.Gly245Arg	MH2	(Aubart et al., 2014)	1	Likely pathogenic
6	c.741_742delAT	p.Thr247Profs*61	MH2	(van de Laar et al., 2011)	1	Pathogenic
6	c.742T > C	p.Phe248Leu	MH2	(Aubart et al., 2014)	1	Likely pathogenic
**6**	**c.748G > A**	**p.Ala250Thr**	**MH2**	**Current study**	**1**	**Likely pathogenic**
**6**	**c.762delC**	p.Met255*	**MH2**	**Current study**	**1**	**Pathogenic**
**6**	**c.772G > C**	**p.Asp258His**	**MH2**	**Current study**	**1**	**Likely pathogenic**
6	c.782C > T	p.Thr261Ile	MH2	(van de Laar et al., 2011)	1	Pathogenic
6	c.788C > T	p.Pro263Leu	MH2	(van de Laar et al., 2012)	1	VUS
6	c.797C > A	p.Ser266*	MH2	(Haller et al., 2015)	1	Pathogenic
**6**	**c.803G > T**	**p.Arg268Leu**	**MH2**	**(** **Schubert et al.,** 2016 **), Current study**	**2**	**Likely pathogenic**
6	c.836G > A	p.Arg279Lys	MH2	(Regalado et al., 2011)	2	Likely pathogenic
6	c.859C > T	p.Arg287Trp	MH2	(van de Laar et al., 2011), (Campens et al., 2015)	2	Likely pathogenic
**6**	**c.860G > A**	**p.Arg287Gln**	**MH2**	**(** **Aubart et al.,** 2014 **), Current study**	**2**	**Likely pathogenic**
**6**	**c.861delG**	p.Arg288Aspfs*53	**MH2**	**Current study**	**1**	**Pathogenic**
7	c.862_871+1dupAGACACATCGG	p.Arg292Aspfs*53	MH2	(Aubart et al., 2014)	1	Pathogenic
7	c.887T > C	p.Leu296Pro	MH2	(Campens et al., 2015)	1	Likely pathogenic
7	c.988A > G	p.Thr330Ala	MH2	(Ye et al., 2013)	1	Likely pathogenic
**7**	**c.1009+1G > A**		**MH2**	**Current study**	**1**	**Pathogenic**
7	c.1009+2T > A		MH2	(Nevidomskyte et al., 2017)	1	Pathogenic
8	c.1045G > C	p.Ala349Pro	MH2	(van de Laar et al., 2012)	1	Likely pathogenic
**8**	**c.1080dupT**	p.Glu361*	**MH2**	**Current study**	**1**	**Pathogenic**
8	c.1081G > T	p.Glu361*	MH2	(van de Laar et al., 2012)	1	Pathogenic
**8**	**c.1091A > G**	**p.Tyr364Cys**	**MH2**	**Current study**	**1**	**Likely pathogenic**
**8**	**c.1102C > T**	p.Arg368*	**MH2**	**(** **Aubart et al.,** 2014 **), Current study**	**2**	**Pathogenic**
**8**	**c.1141G > C**	**p.Gly381Arg**	**MH2**	**Current study**	**1**	**Likely pathogenic**
9	c.1155‐2A > G		MH2	(Campens et al., 2015)	1	Pathogenic
9	c.1170_1179del	p.Ser391Alafs*7	MH2	(Wischmeijer et al., 2013)	1	Pathogenic
9	c.1179_1180dupC	p.Cys394Leufs*4	MH2	(Aubart et al., 2014)	1	Pathogenic
**9**	**c.1185G > C**	**p.Trp395Cys**	**MH2**	**Current study**	**1**	**VUS**
**9**	**c.1199T > C**	**p.Leu400Pro**	**MH2**	**Current study**	**1**	**VUS**
9	c.1208C > T	p.Pro403Leu	MH2	(Martens et al., 2013)	1	Likely pathogenic
**9**	**c.1222G > C**	**p.Asp408His**	**MH2**	**Current study**	**1**	**Likely pathogenic**
**9**	**c.1224C > A**	**p.Asp408Glu**	**MH2**	**Current study**	**1**	**Likely pathogenic**
**9**	**c.1243G > C**	**p.Gly415Arg**	**MH2**	**Current study**	**1**	**Likely pathogenic**
**9**	**c.1247C > T**	**p.Ser416Phe**	**MH2**	**Current study**	**1**	**Likely pathogenic**
9	c.1259G > A	p.Arg420His	MH2	(Proost et al., 2015)	1	VUS
**9**	**c.1265C > T**	**p.Ser422Phe**	**MH2**	**Current study**	**1**	**Likely pathogenic**
9	c.1267A > G	p.Ser423Gly	MH2	(Aubart et al., 2014)	1	Likely pathogenic
**9**	**c.1268G > A**	**p.Ser423Asn**	**MH2**	**Current study**	**1**	**Likely pathogenic**
**9**	**c.1274C > T**	**p.Ser425Phe**	**MH2**	**Current study**	**1**	**VUS**
Deletion from exon 2 onwards: Chr2.hg19: g.(67,408,242)_(67,603,013)del				(Hilhorst‐Hofstee et al., 2013)	1	Pathogenic
**Deletion 15q22.33**				**Current study**	**1**	**Pathogenic**

Mutations identified in this study are indicated in bold.

**Table 2:** Previously described and novel *TGFB2* gene mutations

Exon	c‐Notation	p‐Notation	Domain	Reference	Times reported	Effect
**1**	**c.236A > T**	**p.Gln79Leu**	**LAP**	**Current study**	**1**	**Likely pathogenic**
**1**	**c.244G > T**	**p.Ala82Ser**	**LAP**	**Current study**	**1**	**VUS**
**1**	**c.274G > T**	p.Glu92*	**LAP**	**Current study**	**1**	**Pathogenic**
1	c.294_308delCTACGCCAAGGAGGT	p.Ala100_Tyr104del	LAP	(Lindsay et al., 2012)	1	Likely pathogenic
1	c.297 > A	p.Tyr99*	LAP	(Lindsay et al., 2012)	1	Pathogenic
1	c.304G > T	p.Glu102*	LAP	(Boileau et al., 2012)	1	Pathogenic
**1**	**c.305_307delAGG**	**p.Glu102del**	**LAP**	**Current study**	**1**	**Likely pathogenic**
**3**	**c.475C > T**	p.Arg159*	**LAP**	**(** **Renard et al.,** 2013 **), Current study**	**3**	**Pathogenic**
**3**	**c.518_519 insT**	p.Lys174Glufs*18	**LAP**	**Current study**	**1**	**Pathogenic**
**3**	**c.577C > T**	**p.Arg193Trp**	**LAP**	**(** **Campens et al.,** 2015 **), Current study**	**2**	**Likely pathogenic**
**4**	**c.673G > T**	p.Glu225*	**LAP**	**Current study**	**1**	**Pathogenic**
5	c.687C > A	p.Cys229*	LAP	(Boileau et al., 2012)	1	Pathogenic
5	c.839‐1G > A	p.Gly280Aspfs*41	LAP	(Ritelli et al., 2014)	1	Pathogenic
6	c.873_888dup	p.Asn297*	LAP	(Boileau et al., 2012)	1	Pathogenic
6	c.979C > T	p.Arg327Trp	RKKR motif	(Lindsay et al., 2012), (Renard et al., 2013), (Schubert et al., 2016)	3	Pathogenic
**6**	**c.980G > A**	**p.Arg327Gln**	**RKKR motif**	**(** **Renard et al.,** 2013 **), (** **Campens et al.,** 2015 **), Current study**	**5**	**Pathogenic**
**6**	**c.988C > T**	**p.Arg330Cys**	**RKKR motif**	**(** **Lindsay et al.,** 2012 **), (** **Campens et al.,** 2015 **), Current study**	**4**	**Pathogenic**
**6**	**c.1000delG**	p.Ala334Arg*fs25	**Cytokine**	**Current study**	**1**	**Pathogenic**
7	c.1021_1025delTACAA	p.Tyr341Cysfs*25	Cytokine	(Boileau et al., 2012)	1	Pathogenic
**7**	**c.1042C > T**	**p.Arg348Cys**	**Cytokine**	**(** **Gago‐Diaz et al.,** 2014 **), Current study**	**2**	**Likely pathogenic**
7	c.1097C > A	p.Pro366His	Cytokine	(Lindsay et al., 2012)	1	Likely pathogenic
7	c.1106_1110delACAAT	p.Tyr369Cysfs*26	Cytokine	(Lindsay et al., 2012)	1	Pathogenic
7	c.1125delT	p.Gly376Glufs*17	Cytokine	(Renard et al., 2013)	1	Pathogenic
7	c.1165dupA	p.Ser389Lysfs*8	Cytokine	(Leutermann et al., 2014)	1	Pathogenic
**7**	**c.1170+1G > A**		**Cytokine**	**Current study**	**1**	**Pathogenic**
**8**	**c.1234G > C**	**p.Asp412His**	**Cytokine**	**Current study**	**1**	**VUS**
Entire gene Chr1.hg19: g.(215,588,712)_(222,145,072)del				(Lindsay et al., 2012)	1	Pathogenic
Entire gene Chr1.hg19:g.(216,672,181)_(220,202,575)del				(Lindsay et al., 2012)	1	Pathogenic
Entire gene Chr1.hg19: g.(214,271,966)_(219,506,825)del				(Fontana et al., 2014)	1	Pathogenic
**Entire gene Chr1.hg19:g.(215,713,272)_(218,899,968)del**				**Current study**	**1**	**Pathogenic**

Mutations identified in this study are indicated in bold.

**Table 3:** Previously described and novel *TGFB3* and *SMAD2* gene mutations

Gene	Exon	c‐notation	p‐notation	Domain	Reference	Times reported	Effect
***TGFB3***	**1**	**c.106A > T**	p.Lys36*	**LAP**	**Current study**	**1**	**Pathogenic**
***TGFB3***	**2**	**c.437delT**	p.Leu146Hisfs*68	**LAP**	**Current study**	**1**	**Pathogenic**
*TGFB3*	4	c.704delA	p.Asn235Metfs*11	LAP	(Bertoli‐Avella et al., 2015)	1	Pathogenic
*TGFB3*	4	c.754+2T > C	p.Glu216_Lys251del	LAP	(Bertoli‐Avella et al., 2015)	1	Pathogenic
***TGFB3***	**5**	**c.787G > C**	**p.Asp263His**	**LAP**	**(** **Bertoli‐Avella et al.,** 2015 **), Current study**	**3**	**Likely pathogenic**
***TGFB3***	**5**	**c.796C > T**	**p.Arg266Cys**	**LAP**	**Current study**	**1**	**VUS**
***TGFB3***	**5**	**c.898C > T**	**p.Arg300Trp**	**RKKR motif**	**(** **Bertoli‐Avella et al.,** 2015 **), Current study**	**5**	**Pathogenic**
***TGFB3***	**5**	**c.899G > A**	**p.Arg300Gln**	**RKKR motif**	**(** **Matyas et al.,** 2014 **), Current study**	**2**	**Pathogenic**
*TGFB3*	5	c.898C > G	p.Arg300Gly	RKKR motif	(Kuechler et al., 2015)	1	Likely pathogenic
*TGFB3*	6	c.965T > C	p.Ile322Thr	Cytokine	(Bertoli‐Avella et al., 2015)	1	Likely pathogenic
***TGFB3***	**6**	**c.979G > T**	**p.Asp327Tyr**	**Cytokine**	**Current study**	**1**	**Likely pathogenic**
*TGFB3*	6	c.1095C > A	p.Tyr365*	Cytokine	(Bertoli‐Avella et al., 2015)	1	Pathogenic
*TGFB3*	7	c.1157delT	p.Leu386Argfs*21	Cytokine	(Bertoli‐Avella et al., 2015)	1	Pathogenic
***TGFB3***	**7**	**c.1202T > C**	**p.Leu401Pro**	**Cytokine**	**(** **Bertoli‐Avella et al.,** 2015 **), Current study**	**2**	**Likely pathogenic**
*TGFB3*	7	c.1226G > A	p.Cys409Tyr	Cytokine	(Rienhoff et al., 2013)	1	Pathogenic
***SMAD2***	**8**	**c.954T > A**	**p.Asn318Lys**	**MH2**	**Current study**	**1**	**Likely pathogenic**
***SMAD2***	**9**	**c.1082A > C**	**p.Asn361Thr**	**MH2**	**Current study**	**1**	**Likely pathogenic**
*SMAD2*	10	c.1163A > G	p.Gln388Arg	MH2	(Micha et al., 2015)	1	Likely pathogenic
***SMAD2***	**10**	**c.1190C > A**	**p.Ser397Tyr**	**MH2**	**Current study**	**1**	**Likely pathogenic**
*SMAD2*	11	c.1346T > C	p.Leu449Ser	MH2	(Micha et al., 2015)	1	Likely pathogenic
*SMAD2*	11	c.1369G > A	p.Gly457Arg	MH2	(Micha et al., 2015)	1	Likely pathogenic
***SMAD2***	**11**	**c.1400C > T**	**p.Ser467Leu**	**MH2**	**Current study**	**1**	**VUS**

Mutations identified in this study are indicated in bold.

**Table 4:** Pathogenic variants in *SMAD2*, *SMAD3*, *TGFB2*, and *TGFB3* present in ExAC

Gene	Variant (p‐notation)	Times in ExAC
*SMAD2*	p.Gln388Arg	1/121108
*SMAD3*	p.Arg420His	1/120914
*TGFB2*	p.Arg193Trp	1/121376
*TGFB3*	p.Ile322Thr	1/121368
	p.Asp327Tyr	1/121378

**Figure 3:**
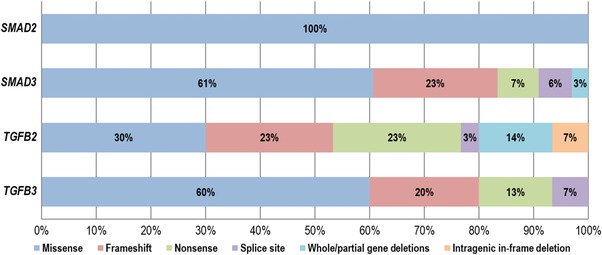
Frequencies of the different types of *SMAD2*, *SMAD3*, *TGFB2*, and *TGFB3* mutations identified so far

For *SMAD3*, 40 variants were previously reported (Aubart et al., 2014; Berthet, Hanna, Giraud, & Soubrier, 2015; Blinc et al., 2015; Burke, Shalhub, & Starnes, 2015; Campens et al., 2015; Courtois et al., 2017; Fitzgerald, Bhat, Conard, Hyland, & Pizarro, 2014; Garcia‐Bermudez, Moustafa, Barros‐Membrilla, & Tizon‐Marcos, 2017; Haller et al., 2015; Hilhorst‐Hofstee et al., 2013; Martens et al., 2013; Nevidomskyte et al., 2017; Proost et al., 2015; Regalado et al., 2011; Schubert, Landis, Shikany, Hinton, & Ware, 2016; van de Laar et al., 2011; van de Laar et al., 2012; van der Linde et al., 2012; Wischmeijer et al., 2013; Yao et al., 2003; Ye et al., 2013; Zhang et al., 2015), whereas 38 probands were identified in this study, with 27 mutations that have never been reported (Table 1 and Figure 1). These *SMAD3* mutations include 61% missense mutations, 23% frameshift mutations, 7% nonsense mutations, 6% splice site mutations, and 3% whole or partial gene deletions (Figure 3). The 30 *TGFB2* mutations include 20 previously published pathogenic variants (Boileau et al., 2012; Campens et al., 2015; Fontana et al., 2014; Gago‐Diaz et al., 2014; Leutermann et al., 2014; Lindsay et al., 2012; Renard et al., 2013; Ritelli et al., 2014; Schubert et al., 2016) and 10 newly identified mutations (Table 2 and Figure 2). In five new probands, previously reported *TGFB2* mutations were identified. New mutations include 30% missense mutations, 23% frameshift mutations, 23% nonsense mutations, 14% whole or partial gene deletions, 7% intragenic in‐frame deletions, and 3% splice site mutations (Figure 3). For *SMAD2* and *TGFB3*, far fewer mutations have been reported (Table 3) (Bertoli‐Avella et al., 2015; Kuechler et al., 2015; Matyas, Naef, Tollens, & Oexle, 2014; Micha et al., 2015; Rienhoff et al., 2013). In addition to the three previously reported *SMAD2* mutations, we identified four new missense mutations, bringing the total to seven missense mutations identified so far (Micha et al., 2015). For *TGFB3*, we identified four additional probands with a mutation that was previously published and four new mutations. The majority of the *TGFB3* mutations consist of missense mutations (60%), whereas 20% are frameshift mutations, 13% are nonsense mutations, and 7% of mutations affect a splice site.

No mutational hotspot is observed for *SMAD2* and *SMAD3* mutations, but the majority (100% and 63%) reside in the MH2 domain, a well conserved region responsible for the oligomerization of SMAD2 or SMAD3 with SMAD4 and subsequent Smad‐dependent activation of downstream transcription (Figure 1). For *TGFB2*, 23% of mutations are located in the exons coding for the active TGF‐β2 cytokine and 28% reside in the RKKR‐motif, a proteolytic cleavage site responsible for releasing mature TGF‐β2 from the latency‐associated peptide (LAP). Of the 17 mutations so far identified in the LAP domain of *TGFB2*, 76% are nonsense or frameshift mutations, whereas 24% are missense mutations. For *TGFB3*, 35% of mutations affect the RKKR‐motif. Remarkably, of the 23 currently known *TGFB3* mutations, three substitute histidine for aspartic acid at amino acid position 263. This arginine belongs to the RGD‐motif that mediates binding to integrins and subsequent integrin‐mediated activation (release) of TGFβ3 from its latent complex (Shi et al., 2011). Since all described patients with this mutation originate from the same county, this might represent a founder mutation (a hypothesis currently under investigation).

## 3 BIOLOGICAL RELEVANCE

For a long time, it was thought that MFS, which is caused by mutations in the fibrillin‐1 gene, and related connective tissue disorders, was caused by a pure structural deficiency of the extracellular matrix (ECM), leading to tissue weakening of the aortic wall and resulting in an increased risk of aneurysm progression and tear. Although some manifestations of MFS, such as lens dislocation and aortic aneurysm could be explained in this way, loss of structural tissue integrity could not easily reconcile the skeletal overgrowth phenotype observed in MFS patients or the low muscle mass and fat stores characteristic of this condition. Mouse models, like *Fbn1^+/C1039G^*, have significantly changed our understanding of disease pathogenesis. It has been shown that altered TGF‐β signaling plays an important role in the lung, valve, skeletal muscle, and aortic pathology of fibrillin‐1‐deficient mice (Cohn et al., 2007; Habashi et al., 2006; Neptune et al., 2003; Ng et al., 2004), indicating that fibrillin‐1 is not only a structural component of the ECM but also a key regulator of TGF‐β signaling (Dallas, Miyazono, Skerry, Mundy, & Bonewald, 1995; Isogai et al., 2003). This new hypothesis was confirmed by a key experiment in which the mutant phenotype in fibrillin‐1‐deficient mice could be attenuated by the administration of TGF‐β‐ neutralizing antibodies (Cohn et al., 2007; Habashi et al., 2006; Neptune et al., 2003; Ng et al., 2004). Shortly after this, the central role of TGF‐β dysregulation was proven by the identification of mutations in the *TGFBR1* and *TGFBR2* genes as the cause of LDS. The effect of these mutations on the TGF‐β pathway is, however, complex as heterozygous loss‐of‐function mutations associate with paradoxical activation of TGF‐β signaling, which was demonstrated by increased phosphorylation of Smad proteins and increased output of TGF‐β‐driven genes.

### 3.1 TGF‐β pathway

TGF‐β is the prototype of a family of secreted polypeptide growth factors essential in development, differentiation, cell growth, migration, apoptosis, and ECM production (Derynck & Akhurst, 2007; Massague, Blain, & Lo, 2000). In humans, three TGF‐β ligand isoforms exist, TGF‐β1, TGF‐β2, and TGF‐β3, encoded by the *TGFB1*, *TGFB2*, and *TGFB3* genes, respectively. TGF‐β is secreted from cells as part of a large latent complex, that consists of the mature TGF‐β cytokine, a dimer of its processed amino terminal propeptide (LAP) and one of three latent TGFβ binding protein‐isoforms (LTBP1, 3, or 4). The latter binds to ECM components such as fibronectin or microfibrils composed of fibrillin‐1. Upon release from this complex, TGF‐β binds to the type II TGF‐β receptor subunit (TGFβR2), which alters its conformation and phosphorylates the type I TGF‐β receptor subunit (TGFβR1), also known as activin receptor‐like kinase 5 (ALK5). In the canonical pathway, TGFβRI then phosphorylates SMAD2 and SMAD3 proteins that transmit the TGFβ signal to the nucleus upon association with SMAD4. SMADs are transcription factors that partner with other factors that drive TGFβ‐mediated gene transcription (Derynck & Zhang, 1996; Feng & Derynck, 2005; Massague et al., 2005; Zhang, 2009). Alternatively, ligand‐activated TGFβ receptors can also initiate non‐canonical signaling cascades, including RhoA and the mitogen‐activated protein kinases ERK, JNK, and p38 (Derynck & Zhang, 2003; Lee et al., 2007; Yamashita et al., 2008).

One of the most unambiguous indications that TGF‐β signaling plays a major role in aneurysm pathogenesis was provided by the finding that *TGFBR1/2* mutations result in LDS. Aortic wall tissue of *TGFBR1* or 2 mutant patients showed increased nuclear accumulation of phosphorylated SMAD2 (pSMAD2) and enhanced expression of TGF‐β driven gene products such as connective tissue growth factor (CTGF), illustrating enhanced TGF‐β signaling. The latter is paradoxical as most of the *TGFBR1* or *2* mutations are predicted to lead to a decrease of the serine‐threonine kinase activity of the receptor (Loeys et al., 2005). An analogous observation was made in medial vascular smooth muscle cells (VSMC) of patients with a *SMAD3* mutation (van de Laar et al., 2011). Finally, in accordance with the findings for *SMAD3*, increased pSMAD2 and CTGF expression was observed in the aortic media of patients with either loss‐of‐function *TGFB2* or *TGFB3* mutations, further confirming the paradoxical observation of increased TGF‐β signaling *in vivo* (Bertoli‐Avella et al., 2015; Lindsay et al., 2012).

In addition to increased expression of pSMAD2 and TGF‐β‐driven gene products, alternative expression of TGFβ‐ligands is observed in LDS patients as well. In human TGFB2 or TGFB3‐deficient aortas, an increase in *TGFB1* expression was observed (Bertoli‐Avella et al., 2015; Lindsay et al., 2012). An analogous observation was made in aneurysmal aortic media of *SMAD3* mutation positive patients (van de Laar et al., 2011). Aortic wall tissue of these patients also revealed an increase in cytoplasmic and nuclear total SMAD3 immunostaining, indicating that other compensatory mechanisms lead to further dysregulation of the pathway (van de Laar et al., 2011). *TGFB2* mutation positive patients showed, besides an increase in *TGFB1*, also an enhanced expression of *TGFB2* in their aortic wall tissue (Boileau et al., 2012). Although *TGFB2* mutations are predicted to result in haploinsufficiency, the exact mechanisms on how loss‐of‐function mutations lead to a paradoxical activation of TGF‐β signaling remain elusive.

Different hypotheses have arisen to address this issue but further experimental validation is mandatory. The first hypothesis states that a potential cell‐autonomous mechanism results in upregulation of TGF‐β signaling (Lindsay & Dietz, 2011). TGF‐β ligand can activate both the canonical and non‐canonical pathway. By a negative feedback loop, the canonical pathway can regulate TGF‐β pathway activity. Mutations in LDS genes (*TGFBR1/2, SMAD3, TGFB2/3*) cause a decrease in canonical signaling hereby downregulating the feedback inhibition in order to restore the canonical signaling pathway. This will result in increased ligand expression and excessive activation of the non‐canonical signaling pathway. Further supporting evidence for the contribution of non‐canonical signaling came from the observation that treatment with a specific ERK inhibitor, RDEA119, abrogates pathological aortic root growth in an MFS mouse model hereby rescuing the aortic aneurysm phenotype (Holm et al., 2011; Habashi et al., 2011). Secondly, as shown by increased expression of TGFB1, both in *TGFB2‐*deficient patients and mice, a shift in the use of specific TGF‐β cytokine isoforms might contribute to the upregulation of the TGF‐β cascade (Lindsay et al., 2012). Thirdly, a non‐cell autonomous effect (paracrine) offers an alternative explanation for the observed increased TGFB1 expression and concomitant enhanced TGF‐β signaling. The VSMC of the thoracic aorta have different embryonic origins: the cells of the ascending aorta are derived from the second heart field and cardiac neural crest, whereas the cells in the descending aorta originate from the somatic mesoderm (proximal descending aorta) or splanchnic mesoderm (distal descending aorta) (Majesky, 2007). Aneurysms can occur throughout the aorta, implicating that there is not a common origin for all VSMCs at sites prone to aneurysm formation. However, aneurysm formation always occurs at locations where cells of divergent origins can interact (at the transition regions) (Gallo et al., 2014; Lindsay & Dietz, 2011). Because cells from distinct origins respond in different ways to TGF‐β stimulation, it is likely that cells of one lineage are more prone to perturbed TGF‐β signaling compared to cells of another origin (Topouzis & Majesky, 1996). Cell types that are more sensitive towards heterozygous LDS mutations might attempt to compensate for the initial loss in TGF‐β signaling by secreting excessive amounts of TGF‐β ligand in their direct environment, leading to overdrive of TGF‐β signaling in neighboring cells which are intrinsically less vulnerable to LDS mutations (Gallo et al., 2014). Finally, since increased expression of pSMAD2 is observed in the aortic media of LDS patients, this might indicate that other TGF‐β related pathways such as angiotensin II or activin signaling cascades are involved as well (Bernard, 2004; Rodriguez‐Vita et al., 2005).

### 3.2 Mouse models

For *SMAD3*, *TGFB2*, and *TGFB3*, multiple mouse models have been developed through the years confirming the hypotheses about the molecular and cellular mechanisms underlying LDS pathogenesis. Mouse models for *SMAD2* have also been developed but *Smad2* knockout mice embryos die before E8,5, due to defective egg cylinder elongation and germ layer formation, which entangles aortic size examination (Waldrip, Bikoff, Hoodless, Wrana, & Robertson, 1998; Weinstein et al., 1998).

Different *Smad3* mouse models initially confirmed the importance of *SMAD3* and the TGF‐β pathway for cartilage integrity. Homozygous mutant mice in which *Smad3* exon 8 is targeted (*Smad3* ^ex8/ex8^) develop progressive degenerative cartilage resembling human osteoarthritis (Yang et al., 1999). Additionally, *Smad3* knock‐out mice (*Smad3* ^−/−^) show phenotypes similar to human osteoarthritis (Li et al., 2009). However, initially, these mouse models were not reported to develop a vascular phenotype (Bonniaud et al., 2004; Li et al., 2009). After identification of *SMAD3* as an aortic aneurysm disease causing gene, the vascular phenotype of the *Smad3* knock‐out mice was studied in more detail (Ye et al., 2013). Necropsy of the *Smad3^−/−^* mice revealed that the majority died from a ruptured aneurysm. Additionally, ultrasound imaging of these mutant mice revealed progressive aortic root and ascending aortic dilation as early as 2 months.

Sanford *et al* reported that *Tgfb2*‐null mice died shortly after birth and had small, thin walled ascending aortas in addition to other developmental defects involving different organ systems (Sanford et al., 1997). In a later study, Bartram et al. (2001) also observed that *Tgfb2*‐null mice die during gestation due to congenital heart disease and display aortic anomalies. Therefore, Lindsay et al. studied the *Tgfb2* heterozygous knockout mouse model to study aneurysm formation in more detail. *Tgfb2^+/−^* mice showed dilatations of the aortic annulus and aortic root, a pattern similar to that of LDS and MFS patients (Loeys et al., 2005; Mc Kusick, 1955), confirming that loss‐of‐function of one *Tgfb2* allele is sufficient to cause aortic root aneurysm. As Western blot of *Tgfb2^+/−^* mouse aortas showed a similar increase in phosphorylation of Smad2/3 and Erk1/2, recapitulating the observations in the human aorta, it can be concluded that increased activation of TGF‐β signaling underlies the observed phenotype (Lindsay et al., 2012). Besides *Tgfb2^+/‐^* mice, the aortas of double‐heterozygous knock‐out mice, *Tgfb2^+/−^*; *Fbn1^C+/C1039G^*, were examined as well. The aortic root dimensions of *Tgfb2^+/−^*; *Fbn1^C+/C1039G^* were significantly increased compared with the wild‐type mice at 2 and 4 months of age. Also elastic fiber fragmentation, higher collagen deposition and increased nuclear accumulation of pSmad2 in the aortic media were observed. *Tgfb1* mRNA levels in the proximal aorta of *Tgfb2^+/−^*; *Fbn1^+/C1039G^* mice at 2 months of age were elevated, which is similar to the observations in human. This is again underlining the paradoxical TGF‐β pathway activation and stressing the complex pathogenesis that goes together with TGF‐β dysregulation.

In 1995, both Proetzel et al. (1995) and Kaartinen et al. (1995) generated, independently, a *Tgfb3* null mutant mouse model. Homozygous *Tgfb3* knockout mice died within 20–24 hr of birth displaying defective palatogenesis but no other concomitant craniofacial or growth abnormalities. Additionally, abnormal pulmonary development was observed. Azhar et al. (2003) described irregularities in the curvature and position of the aortic arches in *Tgfb3* knockout mice, but no major cardiac defects have been reported. More recently, Doetschman et al. (2012) developed *Tgfb3* conditional knockout mice that died at birth from cleft palate defects, similar to *Tgfb3^−/−^* mice, but there was no record of cardiovascular abnormalities in these mice. Further evaluation of the aortic sizes of *Tgfb3* conditional knockout or haploinsufficient mice definitely offer some interesting and necessary research prospects.

## 4 CLINICAL AND DIAGNOSTIC RELEVANCE

The most important clinical finding in LDS patients is dilatation of the aortic root at the level of the sinuses of Valsalva, a feature that nearly all LDS patients will develop ultimately. Aneurysms of the ascending or descending aorta are less frequently observed. Dissection and rupture of these aortic aneurysms tend to occur at a younger age and at smaller diameters in LDS patients compared to MFS patients (Loeys et al., 2006). The aortic phenotype in patients with *SMAD3* mutations is very similar to *TGFBR1/2* patients, whereas *TGFB2* and *TGFB3* cardiovascular features tend to be milder, although severe aortic presentation at young age has also been observed. Non‐penetrance seems more common in *TGFB2/3* families. Using CT or MRI, 3D reconstruction of images from the head to pelvis is needed to identify arterial tortuosity, present in most individuals with a *TGFBR1/2*, *TGFB2*, or *SMAD3* mutation, and aneurysms in the rest of the arterial tree. This is essential because aneurysms distant from the aortic root can be easily overlooked using echocardiography.

Skeletal features in LDS include joint hyperlaxity, scoliosis, arachnodactyly, pectus deformity, and club foot, showing some overlap with those of MFS. Additionally, osteoarthritis and hernia (mostly inguinal) have been frequently observed in all LDS types (Table 5). Although a significant overlap exists between the phenotypic characteristics of *SMAD2/3*, *TGFB2/3* patients and prior *TGFBR1/2* reported features, some differences seem to emerge (Table 5). For example cervical spine instability has not yet been seen in *TGFB2/3* mutations patients, and craniosynostosis has only been reported once in a *SMAD3* patient. For the first time, we report on cleft palate in patients with *TGFB2* mutations. The number of patients with *SMAD2* and *TGFB3* mutations is probably too low to draw any significant conclusions so far. For *TGFB2* and *SMAD3*, a detailed summary of the clinical manifestations, both of the patients previously described in the literature and our newly reported patients, can be found in Suppl. Tables S2 and S3. For *TGFB3* and *SMAD2*, detailed clinical features of all patients reported so far are listed in Suppl. Tables S4 and S5.

**Table 5:** Comparison of the clinical manifestations of patients with *SMAD2*, *SMAD3*, *TGFB2*, and *TGFB3* mutations

	TGFB2	TGFB3	SMAD3	SMAD2
Hypertelorism	+	+	+	+
Bifid uvula/cleft palate	+	+	+	−
Exotropia	+	+	+	?
Craniosynostosis	−	−	+	?
Cervical spine instability	−	+	+	?
Retrognathia	+	+	+	−
Scoliosis	+	+	+	+
Club foot	+	+	+	−
Osteo‐arthritis	+	+	+	+
Dural ectasia	+	?	+	+
Pneumothorax	+	−	+	+
Hernia	+	+	+	+
Dissection at young age	+	?	+	−
Arterial tortuosity	+	−	+	+

+ indicates presence of the clinical feature, − indicates absence of the clinical features and a question mark illustrates presence of the clinical feature is unknown.

Although no formal diagnostic criteria have been developed, genetic testing of the LDS genes should be considered in the following scenarios: (1) patients with the typical clinical trial of hypertelorism, cleft palate/bifid uvula and arterial tortuosity/aneurysm; (2) early onset aortic aneurysm with variable combination of other features including arachnodactyly, camptodactyly, club feet, craniosynostosis (all types), blue sclerae, thin skin with atrophic scars, easy bruising, joint hypermobility, bicuspid aortic valve, patent ductus arteriosus, atrial and ventricular septum defects; (3) sporadic young probands with aortic root dilatation/dissection; (4) families with autosomal dominant thoracic aortic aneurysms, especially those families with early onset aortic/arterial dissection, aortic disease beyond the aortic root (including cerebral arteries); (5) patients with a MS‐like phenotype, especially those without ectopia lentis, but with aortic and skeletal features not fulfilling the MS diagnostic criteria; (6) patients with clinical features reminiscent of vascular Ehlers–Danlos syndrome (thin skin with atrophic scars, easy bruising, joint hypermobility) and normal type III collagen biochemistry and/or normal *COL3A1* genetic testing.

The diagnosis of LDS is established in a proband (by definition a person without a known family history of LDS), who has a heterozygous pathogenic variant in *SMAD2*, *SMAD3*, *TGFB2*, *TGFB3, TGFBR1*, or *TGFBR2* and either of the following (MacCarrick et al., 2014): (1) aortic root enlargement (defined as an aortic root *z*‐score greater than or equal to 2.0) or type A dissection or (2) compatible systemic features including characteristic craniofacial, skeletal, cutaneous and/or vascular manifestations found in combination. Special emphasis is given to arterial tortuosity, prominently including the head and neck vessels, and to aneurysms or dissections involving medium‐to‐large muscular arteries throughout the arterial tree. In the presence of a family history of documented LDS, the diagnosis can be made in at‐risk relatives on the basis of molecular genetic testing, even if vascular involvement or other features are not yet apparent.

## 5 FUTURE PROSPECTS

The recent identification of *SMAD2,3* and *TGFB2,3* as disease causing genes responsible for LDS phenotypes further pinpoints altered TGF‐β signaling as the culprit in aortic aneurysm pathology. As we anticipate that more genes will cause similar clinical LDS phenotypes, there is a clear rationale for the development of gene panels. This makes it possible to screen multiple candidate genes at one go, taking advantage of the next‐generation sequencing techniques, which are more time and cost efficient compared with the elaborate Sanger sequencing technique. As shown by recent studies, this will facilitate the assessment of an accurate genetic diagnosis for LDS patients hereby surely benefiting patient management (Campens et al., 2015; Proost et al., 2015).

Cardiovascular manifestations of LDS can be managed in a medical and/or surgical treatment strategy. Since vascular disease is more aggressive in LDS compared with MFS, prophylactic aortic surgery is already being recommended at smaller aortic root dimensions of 4.0‐4.5 cm (MacCarrick et al., 2014). In order to reduce aortic wall‐shear stress and eventually aortic dilatation, beta‐blockers, such as atenolol, have been the first‐line treatment (Shores, Berger, Murphy, & Pyeritz, 1994). However, since aortic‐root tissue of LDS patients shows excessive TGF‐β activation and signaling, therapies that reduce TGF‐β pathway activation offer attractive therapeutic targets. Indeed, angiotensin II type 1 receptor blockers, such as losartan, reduce the rate of aortic root growth by lowering the expression of TGFβ ligands, receptors, and activators (Everett, Tufro‐McReddie, Fisher, & Gomez, 1994; Fukuda et al., 2000; Habashi et al., 2006; Naito et al., 2004). Although a randomized clinical trial comparing losartan with atenolol did not show a significant difference in the rate of aortic‐root dilatation between these two treatment groups, the observation that aortic‐root *z*‐scores decrease especially in younger patients advocates to start therapy earlier in the disease course (Lacro et al., 2014). Alternatively, the use of ERK inhibitors (RDEA119) and angiotensin II type 2 receptor agonists offer interesting alternative treatment options (Loeys, 2015).

The full spectrum of phenotypes associated with some genes of the TGF‐β related vasculopathies, such as *FBN1* and *TGFBR1/2*, has been described extensively (Arslan‐Kirchner et al., 2011; Cook, Carta, Galatioto, & Ramirez, 2015; Lerner‐Ellis et al., 2014; Ramachandra et al., 2015; Romaniello et al., 2014). For the more recently identified LDS genes (*TGFB2,3* and *SMAD2,3*), the phenotypic spectrum has not been fully studied so far. By summarizing the clinical manifestations of *TGFB2,3* and *SMAD2,3* mutation patients, this manuscript extends the spectrum of phenotypes associated with these LDS genes. However, the screening of larger cohorts of patients with a LDS‐like phenotype, whether or not using gene panels, will be required to reveal the full phenotypic spectrum of LDS.

## Supporting information

Supporting Information Table S1

Supporting Information Table S2

Supporting Information Table S3

Supporting Information Table S4

Supporting Information Table S5
