# Supplementary material for: A mutation update on the LDS‐associated genes TGFB2/3 and SMAD2/3
Source: Hum Mutat. 2018 Mar 6;39(5):621–34. doi: 10.1002/humu.23407 (PMC5947146; doi:10.1002/humu.23407)
Supplement: Supplementary file 1 — Supporting Information Table S1 [file HUMU-39-621-s001.pdf]

Supplementary Table S1: Pathogenicity classification of all *SMAD2/3* and *TGFB2/3* variants described in this paper according to the ACMG guidelines. Evidence is provided by the criteria defined by Richards et al., 2015

Gene	c-notation	p-notation	Effect	Evidence
SMAD2	c.954T>A	p.Asn318Lys	Likely pathogenic (V)	PM2, PP3, PP2, PP1-M
SMAD2	c.1082A>C	p.Asn361Thr	Likely pathogenic (V)	PM2, PP3, PP2, PP1-M
SMAD2	c.1163A>G	p.Gln388Arg	Likely pathogenic (III)	PP3, PS3, PP2
SMAD2	c.1190C>A	p.Ser397Tyr	Likely pathogenic (II)	PM2, PP3, PP2, PP1-S
SMAD2	c.1346T>C	p.Leu449Ser	Likely pathogenic (V)	PM2, PP3, PP2, PP1-M
SMAD2	c.1369G>A	p.Gly457Arg	Likely pathogenic (II)	PM2, PP3, PP2, PS2
SMAD2	c.1400C>T	p.Ser467Leu	VUS	PM2, PP3, PP2
SMAD3	c.2T>C	p.Met1Thr	Pathogenic (Ic)	PM2, PVS1, PP2
SMAD3	c.3G>A	p.Met1Ile	Pathogenic (Ic)	PM2, PVS1, PP2
SMAD3	c.221G>A	p.Arg74Gln	Likely pathogenic (V)	PM2, PM5, PP2, PP1, PP4
SMAD3	c.266G>A	p.Cys89Tyr	Pathogenic (IIIb)	PM2, PP2, PP1-S, PP1-M, PP1, PP4
SMAD3	c.269G>A	p.Arg90His	Likely pathogenic (V)	PM2, PP2, PP1, PP1-M, PP4
SMAD3	c.281G>T	p.Trp94Leu	VUS	PM2, PP2, PP1, PP4
SMAD3	c.290T>G	p.Leu97Arg	VUS	PM2, PP2, PP4
SMAD3	c.300_301insAGGGCCGGCAGGC	p.His101Argfs*14	Pathogenic (Ic)	PM2, PVS1, PP4
SMAD3	c.313delG	p.Ala105Profs*11	Pathogenic (Ic)	PM2, PVS1, PP4
SMAD3	c.335C>T	p.Ala112Val	Likely pathogenic (V)	PM2, PP2, PP1-M, PP4
SMAD3	c.370C>A	p.Pro124Thr	VUS	PM2, PP2, PP5, PP4
SMAD3	c.374A>G	p.Tyr125Cys	Likely pathogenic (V)	PM2, PM5, PP2, PP4
SMAD3	c. 374A>C	p.Tyr125Ser	Likely pathogenic (V)	PM2, PM5, PP2, PP4
SMAD3	c.401-6G>A	p.Val134Aspfs*33	Pathogenic (Ia)	PM2, PVS1, PS1, PP4
SMAD3	c.401_405dup	p.Pro136Phefs*52	Pathogenic (Ic)	PM2, PVS1, PP4
SMAD3	c.455delC	p.Pro152Hisfs*34	Pathogenic (Ic)	PM2, PVS1, PP4
SMAD3	c.511G>T	p.Glu171*	Pathogenic (Ic)	PM2, PVS1, PP4
SMAD3	c.532+1G>C		Pathogenic (Ic)	PM2, PVS1, PP4
SMAD3	c.539_540insC	p.Pro180Thrfs*7	Pathogenic (Ia)	PM2, PVS1, PS1, PP4
SMAD3	c.546delT	p.Gly183Alafs*3	Pathogenic (Ic)	PM2, PVS1, PP4
SMAD3	c.584_585insTC	p.Gln195Hisfs*3	Pathogenic (Ib)	PP4, PVS1, PM2, PM4
SMAD3	c.652delA	p.Asn218Thrfs*23	Pathogenic (Ib)	PM2, PVS1, PM4, PP1-M
SMAD3	c.668delC	p.Pro223Glnfs*18	Pathogenic (Ic)	PM2, PVS1, PP4
SMAD3	c.715G>A	p.Glu239Lys	Pathogenic (II)	PM2, PS1, PP2, PP1-S
SMAD3	c.728G>C	p.Arg243Pro	Likely pathogenic (II)	PM2, PP2, PP3, PP1-S, PP4
SMAD3	c.733G>A	p.Gly245Arg	Likely pathogenic (VI)	PM2, PP3, PP5, PP4, PP2
SMAD3	c.741_742delAT	p.Thr247Profs*61	Pathogenic (Ic)	PM2, PVS1, PP4
SMAD3	c.742T>C	p.Phe248Leu	Likely pathogenic (VI)	PM2, PP3, PP2, PP4, PP5
SMAD3	c.748G>A	p.Ala250Thr	Likely pathogenic (II)	PM2, PP3, PP2, PP1-S
SMAD3	c.762delC	p.Met255*	Pathogenic (Id)	PM2, PVS1, PP4, PP5
SMAD3	c.772G>C	p.Asp258His	Likely pathogenic (VI)	PM2, PP2, PP5, PP4, PP3
SMAD3	c.782C>T	p.Thr261Ile	Pathogenic (IIIc)	PM2, PP3, PP2, PP5, PP1-S, PP4
SMAD3	c.788C>T	p.Pro263Leu	VUS	PM2, PP3, PP2, PP4
SMAD3	c.797C>A	p.Ser266*	Pathogenic (Ic)	PM2, PVS1, PP4
SMAD3	c.803G>T	p.Arg268Leu	Likely pathogenic (II)	PM2, PS1, PP3, PP2, PP4
SMAD3	c.836G>A	p.Arg279Lys	Likely pathogenic (VI)	PM2, PP3, PP2, PP1, PP4
SMAD3	c.859C>T	p.Arg287Trp	Likely pathogenic (II)	PM2, PS1, PP3, PP2, PP4
SMAD3	c.860G>A	p.Arg287Gln	Likely pathogenic (II)	PM2, PS1, PM5, PP3
SMAD3	c.861delG	p.Arg288Aspfs*53	Pathogenic (Id)	PM2, PVS1, PP3, PP2
SMAD3	c.862_871+1dupAGACACATCGG	p.Arg292Aspfs*53	Pathogenic (Ic)	PM2, PVS1, PP3
SMAD3	c.887T>C	p.Leu296Pro	Likely pathogenic (V)	PM2, PP3, PP2, PP1-M
SMAD3	c.988A>G	p.Thr330Ala	Likely pathogenic (VI)	PM2, PP3, PP1, PP4, PP2
SMAD3	c.1009+1G>A		Pathogenic (Ic)	PM2, PVS1, PP3
SMAD3	c.1009+2T>A		Pathogenic (Ic)	PM2, PVS1, PP3
SMAD3	c.1045G>C	p.Ala349Pro	Likely pathogenic (V)	PM2, PP3, PP2, PP1-M
SMAD3	c.1080dupT	p.Glu361*	Pathogenic (Ia)	PM2, PVS1, PP3, PS1
SMAD3	c.1081G>T	p.Glu361*	Pathogenic (Ia)	PM2, PVS1, PS1
SMAD3	c.1091A>G	p.Tyr364Cys	Likely pathogenic (VI)	PM2, PP3, PP2, PP1, PP4

SMAD3	c.1102C>T	p.Arg368*	Pathogenic (Ia)	PM2, PVS1, PS1
SMAD3	c.1141G>C	p.Gly381Arg	Likely pathogenic (V)	PM2, PP3, PP2, PP1-M
SMAD3	c.1155-2A>G		Pathogenic (Ic)	PM2, PVS1, PP3
SMAD3	c.1170_1179del	p.Ser391Alafs*7	Pathogenic (Ic)	PM2, PVS1, PP3
SMAD3	c.1179_1180dupC	p.Cys394Leufs*4	Pathogenic (Ic)	PM2, PP3, PVS1
SMAD3	c.1185G>C	p.Trp395Cys	VUS	PM2, PP3, PP2
SMAD3	c.1199T>C	p.Leu400Pro	VUS	PM2, PP3, PP2
SMAD3	c.1208C>T	p.Pro403Leu	Likely pathogenic (II)	PM2, PP3, PP2, PS2
SMAD3	c.1222G>C	p.Asp408His	Likely pathogenic (V)	PM2, PM5, PP3, PP2
SMAD3	c.1224C>A	p.Asp408Glu	Likely pathogenic (V)	PM2, PP3, PM5, PP2
SMAD3	c.1243G>C	p.Gly415Arg	Likely pathogenic (V)	PM2, PP3, PP2, PP1-M
SMAD3	c.1247C>T	p.Ser416Phe	Likely pathogenic (V)	PM2, PP3, PP2, PP1-M
SMAD3	c.1259G>A	p.Arg420His	VUS	PP3, PP2
SMAD3	c.1265C>T	p.Ser422Phe	Likely pathogenic (II)	PM2, PP3, PP2, PP1-S
SMAD3	c.1267A>G	p.Ser423Gly	Likely pathogenic (V)	PM2, PP3, PP2, PP1-M
SMAD3	c.1268G>A	p.Ser423Asn	Likely pathogenic (II)	PM2, PP3, PP2, PS2
SMAD3	c.1274C>T	p.Ser425Phe	VUS	PM2, PP3, PP2
SMAD3	Deletion from exon 2 onwards: Chr2.hg19: g.(67,408,242)_(67,603,013)del		Pathogenic (Ic)	PVS1, PP3, PM2
SMAD3	Deletion 15q22.33		Pathogenic (Ic)	PM2, PVS1, PP3
TGFB2	c.236A>T	p.Gln79Leu	Likely pathogenic (VI)	PM2, PP3, PP2, PP1, PP4
TGFB2	c.244G>T	p.Ala82Ser	VUS	PM2, PP3, PP2, PP4
TGFB2	c.274G>T	p.Glu92*	Pathogenic (Ic)	PM2, PVS1, PP1
TGFB2	c.294_308delCTACGCCA AGGAGGT	p.Ala100_Tyr104del	Likely pathogenic (V)	PM2, PP3, PM4, PP1, PP4
TGFB2	c.297 >A	p.Tyr99*	Pathogenic (Id)	PM2, PVS1, PP3, PP1, PP4
TGFB2	c.304G>T	p.Glu102*	Pathogenic (Id)	PM2, PVS1, PP3, PP1, PP4
TGFB2	c.305_307delAGG	p.Glu102del	Likely pathogenic (V)	PM2, PM4, PP3, PP1, PP4
TGFB2	c.475C>T	p.Arg159*	Pathogenic (Ia)	PM2, PVS1, PS1, PP3, PP4
TGFB2	c.518_519 insT	p.Lys174Glufs*18	Pathogenic (Id)	PM2, PVS1, PP3, PP4
TGFB2	c.577C>T	p.Arg193Trp	Likely pathogenic (III)	PP3, PP2, PP1-S, PP4
TGFB2	c.673G>T	p.Glu225*	Pathogenic (Id)	PM2, PVS1, PP3, PP4
TGFB2	c.687C>A	p.Cys229*	Pathogenic (Id)	PM2, PVS1, PP3, PP1, PP4
TGFB2	c.839-1G>A	p.Gly280Aspfs*41	Pathogenic (Id)	PM2, PVS1, PP3, PP1, PP4
TGFB2	c.873_888dup	p.Asn297*	Pathogenic (Id)	PM2, PVS1, PP3, PP1, PP4
TGFB2	c.979C>T	p.Arg327Trp	Pathogenic (II)	PM2, PS1, PM5, PP3, PM1, PP1-S
TGFB2	c.980G>A	p.Arg327Gln	Pathogenic (IIIa)	PM2, PS1, PM5, PP3, PM1, PP1
TGFB2	c.988C>T	p.Arg330Cys	Pathogenic (IIIb)	PM2, PS1, PP3, PM1, PP1
TGFB2	c.1000delG	p.Ala334Arg*fs25	Pathogenic (Ic)	PM2, PVS1, PP3
TGFB2	c.1021_1025delTACAA	p.Tyr341Cysfs*25	Pathogenic (Id)	PM2, PVS1, PP3, PP1
TGFB2	c.1042C>T	p.Arg348Cys	Likely pathogenic (V)	PM2, PP3, PP2, PP1-M
TGFB2	c.1097C>A	p.Pro366His	Likely pathogenic (V)	PM2, PP3, PP2, PP1-M
TGFB2	c.1106_1110delACAAT	p.Tyr369Cysfs*26	Pathogenic (Ia)	PM2, PVS1, PP3, PP1-S
TGFB2	c.1125delT	p.Gly376Glufs*17	Pathogenic (Ic)	PM2, PVS1, PP3
TGFB2	c.1165dupA	p.Ser389Lysfs*8	Pathogenic (Ib)	PM2, PVS1, PP3, PP1-M
TGFB2	c.1170+1G>A		Pathogenic (Id)	PM2, PVS1, PP3, PP1
TGFB2	c.1234G>C	p.Asp412His	VUS	PM2, PP3, PP2, PP4
TGFB2	Entire gene Chr1.hg19: g.(215,588,712)_(222,145,072)del		Pathogenic (Ia)	PM2, PVS1, PS2
TGFB2	Entire gene Chr1.hg19: g.(216,672,181)_(220,202,575)del		Pathogenic (Ia)	PM2, PVS1, PS2
TGFB2	Entire gene Chr1.hg19: g.(214,271,966)_(219,506,825)del		Pathogenic (Ia)	PM2, PVS1, PP3, PS2
TGFB2	Entire gene Chr1.hg19: g.(215,713,272)_(218,899,968)del		Pathogenic (Ia)	PM2, PVS1, PS2
TGFB3	c.106A>T	p.Lys36*	Pathogenic (Ic)	PM2, PVS1, PP3
TGFB3	c.437delT	p.Leu146Hisfs*68	Pathogenic (Ic)	PM2, PVS1, PP3
TGFB3	c.704delA	p.Asn235Metfs*11	Pathogenic (Id)	PM2, PVS1, PP3, PP1
TGFB3	c.754+2T>C	p.Glu216_Lys251del	Pathogenic (Id)	PM2, PVS1, PP3, PP1
TGFB3	c.787G>C	p.Asp263His	Likely pathogenic (II)	PM2, PP3, PS1, PP2, PP1

TGFB3	c.796C>T	p.Arg266Cys	VUS	PM2, PP3, PP1
TGFB3	c.898C>T	p.Arg300Trp	Pathogenic (IIIb)	PM2, PP3, PS1, PP2, PP1-M
TGFB3	c.899G>A	p.Arg300Gln	Pathogenic (IIIa)	PM2, PS1, PP3, PM5, PM1
TGFB3	c.898C>G	p.Arg300Gly	Likely pathogenic (IV)	PM2, PM5, PP3, PM1, PP2, PP1
TGFB3	c.965T>C	p.Ile322Thr	Likely pathogenic (V)	PP3, PP2, PM1, PP1-M, PP1
TGFB3	c.979G>T	p.Asp327Tyr	Likely pathogenic (V)	PP3, PP2, PM1, PP1-M, PP1
TGFB3	c.1095C>A	p.Tyr365*	Pathogenic (Ia)	PM2, PVS1, PP3, PM1, PS2
TGFB3	c.1157delT	p.Leu386Argfs*21	Pathogenic (Ic)	PM2, PVS1, PP3
TGFB3	c.1202T>C	p.Leu401Pro	Likely pathogenic (IV)	PM2, PM5, PP3, PM1, PP2
TGFB3	c.1226G>A	p.Cys409Tyr	Pathogenic (IIIb)	PM2, PP3, PM1, PP2, PS3

VUS = Variant of Unknown Significance
