# Supplementary material for: A mutation update on the LDS‐associated genes TGFB2/3 and SMAD2/3
Source: Hum Mutat. 2018 Mar 6;39(5):621–34. doi: 10.1002/humu.23407 (PMC5947146; doi:10.1002/humu.23407)
Supplement: Supplementary file 2 — Supporting Information Table S2 [file HUMU-39-621-s002.pdf]

Supplementary Table S2: Comparison of the clinical features between previously reported and newly identified *TGFB2* mutation patients.

	(Boileau, et al., 2012)	(Lindsay, et al., 2012)	(Renard, et al., 2013)	(Fontana, et al., 2014)	(Leutermann, et al., 2014)	(Ritelli, et al., 2014)	(Gago-Diaz, et al., 2014)	(Schubert, et al., 2016)	Total literature (%)	Total current study (%)
Abnormal uvula	0/13	3/15							3/28 (11)	5/28 (18)
Aortic aneurysm	4/10	10/15	4/6	1/1	2/2	0/2	3/3	1/1	25/40 (63)	21/33 (64)
Aortic dissection	2/19	1/15	2/6		0/3	0/2			5/45 (11)	3/29 (10)
Aortic repair	4/19	4/15	0/6		2/3	0/2			10/45 (22)	5/29 (17)
Aortic tortuosity										1/15 (7)
Arachnodactyly	8/13	8/15	2/6	0/1	0/2	1/2			19/39 (49)	14/29 (48)
Arterial aneurysm										4/17 (24)
Arterial tortuosity	3/5	1/15	1/1		2/2	1/1		1/1	9/25 (36)	8/20 (40)
Artery dissection										2/5 (40)
Ascending aortic aneurysm										2/17 (12)
Astigmatism		1/15							1/15 (7)	
Atrial septal defect								1/1	1/1 (100)	1/18 (6)
Atrophic scarring										1/17 (6)
Bicuspid aortic valve		2/15					1/3		3/18 (17)	2/25 (8)
Blue sclerae										1/21 (5)
Camptodactyly			1/6					1/1	2/7 (29)	1/19 (0)
Cataract			1/6						1/6 (17)	0/14 (0)
Cervical spine instability										0/17 (0)
Cleft palate	0/13								0/13 (0)	4/22 (18)
Club feet		5/15	1/6	0/1	1/2	0/2			7/26 (27)	3/27 (11)
Craniosynostosis										0/19 (0)
Defective scaring		2/15	1/6			1/2			4/23 (17)	
Dolichocephaly	0/13						1/3		1/16 (6)	6/18 (33)
Dolichostenomelia										10/20 (50)
Downslanting palebral fissures	0/13	10/15	1/6		0/0			1/1	12/35 (34)	16/26 (62)
Dural ectasia	3/5	1/6			0/2	1/1			5/14 (36)	7/12 (58)
Easy bruising		5/11			0/2	2/2			5/15 (33)	11/29 (38)
Ectopia lentis	0/13				0/2				0/15 (0)	0/16 (0)
Emphysema	2/13								2/13 (15)	
Excavation papillae		1/15							1/15 (7)	
Flat cornea	2/8								2/8 (25)	
Fractures										2/15 (13)
Glaucoma										2/16 (13)
Hernia	6/17	10/15			2/2	1/2		1/1	20/37 (54)	9/30 (30)
High arched palate	9/15	10/15	2/6	1/1	1/2	2/2	1/3		26/44 (59)	10/10 (100)
Hyperelastic skin	0/13					2/2			2/15 (13)	
Hyperopia		1/15							1/15 (7)	
Hypertelorism		5/15		1/1					6/16 (38)	10/28 (36)
Inflammatory bowel disease										1/15 (7)
Joint dislocation										4/22 (18)
Joint hypermobility	10/15	7/15	4/6	1/1	2/3	2/2	3/3	1/1	30/46 (65)	22/31 (71)
Lens opacity		1/15							1/15 (7)	
Malar hypoplasia	0/13								0/13 (0)	12/19 (63)
MV insufficiency or prolaps	3/19	7/10	4/6	1/1	0/2	2/2			17/40 (43)	11/33 (33)
Myopia		4/15	3/6	1/1					8/22 (36)	16/26 (62)
Osteo-arthritis										2/15 (13)
Osteoporosis										1/11 (9)
Pectus deformities	7/16	9/15	2/6	1/1	1/3	1/2		1/1	22/44 (50)	18/33 (54)
Pes planus	11/15	8/15	1/6		1/2	0/2	1/3		22/43 (51)	18/30 (60)
Phlebitis	2/18								2/18 (11)	
Pneumothorax	1/17								1/17 (6)	1/18 (6)
Protrusio acetabuli	1/8								1/8 (13)	
Retinal detachment		1/15							1/15 (7)	1/16 (6)
Retrognathia	2/13	11/15	2/6	1/1	2/2			1/1	19/38 (50)	18/29 (62)
Scoliosis	4/15	5/15	1/6	1/1	3/3	2/2	1/3	1/1	18/46 (39)	12/30 (40)
Spondylolisthesis	1/7								1/7 (14)	1/11 (9)
Strabismus			1/6	1/1					2/7 (29)	4/20 (20)
Striae	8/15	3/15	1/6		0/2	1/2			13/40 (33)	11/28 (39)
Tall stature	8/19	11/15	0/1		2/2	1/2			22/39 (56)	
Thin, translucent skin										6/21 (29)
Tooth enamel defect				1/1					1/1 (100)	2/15 (13)
