# Supplementary material for: A mutation update on the LDS‐associated genes TGFB2/3 and SMAD2/3
Source: Hum Mutat. 2018 Mar 6;39(5):621–34. doi: 10.1002/humu.23407 (PMC5947146; doi:10.1002/humu.23407)
Supplement: Supplementary file 3 — Supporting Information Table S3 [file HUMU-39-621-s003.pdf]

Supplementary Table S3: Comparison of the clinical features between previously reported and newly identified *SMAD3* mutation patients.

	(van de Laar, et al., 2011 ; van de Laar, et al., 2012)	(Wischmeijer, et al., 2013)	(Regalado, et al., 2011)	(Hilhorst-Hofstee, et al., 2013)	(Fitzgerald, et al., 2014)	(Martens, et al., 2013)	(Aubart, et al., 2014)	(Burke, et al., 2015)	(Berthet, et al., 2015)	(Haller, et al., 2015)	(Blinc, et al., 2015)	(Zhang, et al., 2015)	(Schubert, et al., 2016)	(Nevidomskyte, et al., 2017)	(Garcia-Bermudez, et al., 2017)	(Courtois, et al., 2017)	Total literature (%)	Total current study (%)
Abdominal aortic aneurysm	4/33		2/42													1/1	7/76 (9)	
Abnormal palate	15/28			2/7					1/1							0/1	18/37 (49)	
Abnormal uvula	13/25	2/3	1/8	3/7						0/1						0/1	19/45 (42)	16/39 (41)
Aneurysm(s) of cerebral arteries	6/16															0/1	6/17 (35)	
Aortic aneurysm	28/39	4/7		5/8	1/1	1/1			1/1	0/2	0/1		1/1				41/61 (67)	17/33 (52)
Aortic dissection/rupture	13/39	1/7	13/42	1/8			13/50	1/1	1/1	0/2	0/2			1/1			44/153 (29)	12/31 (39)
Aortic surgery														1/1		1/1	2/2 (100)	16/33 (49)
Aortic tortuosity	10/26	2/5				1/1						1/1				0/1	14/34 (41)	4/23 (17)
Arachnodactyly	13/33		0/8													0/1	13/42 (31)	9/32 (28)
Arterial aneurysm								1/1			1/1	1/1		1/1		1/1	5/5 (100)	10/27 (37)
Arterial tortuosity				3/5							1/1					0/1	4/6 (67)	12/26 (46)
Arterial tortuosity of cerebral arteries	8/16		2/6														10/22 (45)	
Arterial tortuosity of thoracic/abdominal arteries	8/21		1/17			1/1											10/39 (26)	
Artery dissection														1/1	1/1		2/2 (100)	2/3 (67)
Atrial fibrillation	8/33																8/33 (24)	
Atrophic scars			1/8													0/1	1/9 (11)	7/27 (26)
Baldder/uterus/bowel prolapse	7/17																7/17 (41)	
Bicuspid aortic valve																		3/28 (11)
Blue sclerae																0/1	0/1 (0)	2/29 (7)
Camptodactyly	4/30		1/8													0/1	5/39 (13)	3/29 (10)
Cataract																0/1	0/1 (0)	4/19 (21)
Cervical spine instability																0/1	0/1 (0)	1/22 (5)
Chronic fatigue	11/28 (39)																11/28 (39)	
Cleft palate															0/1	0/1	0/2 (0)	0/34 (0)
Club foot																0/1	0/1 (0)	4/31 (13)
Congenital heart disease	3/33																3/33 (11)	0/26 (0)
Craniosynostosis															0/1	0/1	0/2 (0)	1/31 (3)
Delayed wound healing																0/1	0/1 (0)	1/28 (4)
Dental malocclusion	8/15		0/8														8/23 (35)	
Dolichocephaly																0/1	0/1 (0)	9/28 (32)
Dolichostenomelia	7/33															0/1	7/33 (21)	6/29 (20)
Downslant palpebral fissures															0/1	0/1	0/2 (0)	11/27 (41)
Dural ectasia	7/?	1/2	1/8														9/?	8/16 (50)
Easy bruising	10/28	1/5	2/8													1/1	14/42 (33)	12/29 (41)
Ectopia lentis																0/1	0/1 (0)	0/19 (0)
Eosinophilic esophagitis																0/1	0/1 (0)	0/12 (0)
Exotropia																0/1	0/1 (0)	1/28 (4)
Food allergy																1/1	1/1 (100)	3/15 (20)
Fractures																0/1	0/1 (0)	7/26 (27)
Genu Valgum		1/5															1/5 (20)	
Glaucoma																0/1	0/1 (0)	1/18 (6)
Hernia	17/40	2/5	4/8	2/7		1/1										0/1	26/62 (42)	11/29 (38)
Hiatus hernia		1/5															1/5 (20)	
High arched palate																		2/3 (67)
Hypertelorism	10/32	1/5	0/8	2/7						0/1					0/1	0/1	13/55 (24)	17/34 (50)
Inflammatory bowel disease																0/1	0/1 (0)	0/19 (0)
Intervertebral disc degeneration	34/37	2/3	2/25	4/6													42/71 (59)	
Intracranial aneurysm or subarachnoid hemorrhage			4/42														4/42 (10)	
Joint dislocation															0/1	0/1	0/2 (0)	4/26 (15)
Joint laxity	3/31		4/8	1/7		0/1	4/23						1/1			1/1	14/72 (19)	22/39 (56)

	(van de Laar, et al., 2011 ; van de Laar, et al., 2012)	(Wischmeijer, et al., 2013)	(Regalado, et al., 2011)	(Hilhorst-Hofstee, et al., 2013)	(Fitzgerald, et al., 2014)	(Martens, et al., 2013)	(Aubart, et al., 2014)	(Burke, et al., 2015)	(Berthet, et al., 2015)	(Haller, et al., 2015)	(Blinc, et al., 2015)	(Zhang, et al., 2015)	(Schubert, et al., 2016)	(Nevidomskyte, et al., 2017)	(Garcia-Bermudez, et al., 2017)	(Courtois, et al., 2017)	Total literature (%)	Total current study (%)
Learning difficulties				3/7													3/7 (43)	
Left ventricular hypertrophy	6/33				1/1												7/34 (21)	
Malar Hypoplasia			3/8													0/1	3/9 (33)	11/32 (34)
Meniscal lesions	7/25																7/25 (28)	
Mitral valve anomalies	18/36	2/5	4/23	0/8	1/1												25/73 (34)	9/31 (29)
Myopia																0/1	0/1 (0)	10/29 (35)
Nasal polyps																		0/4 (0)
Osteoarthritis facet and/or uncovertebral joints (spine)	20/26						17/20										37/46 (80)	
Osteoarthritis feet/ankle	8/26			1/6													9/32 (28)	
Osteoarthritis hand/wrist	14/26			2/6													16/32 (50)	
Osteoarthritis hip	4/26			2/6													6/32 (19)	
Osteoarthritis in one or more joints	25/26	0/2	7/25	2/6			13/14	1/1				1/1				0/1	49/76 (64)	16/25 (64)
Osteoarthritis knee	13/26			1/6													14/32 (44)	
Osteochondritis dissecans	14/25			0/6			1/23										15/54 (28)	
Osteoporosis																0/1	0/1 (0)	6/17 (35)
Painful joints	23/27																23/27 (85)	
Patent ductus arteriosus																		1/27 (4)
Pectus deformity	12/33	1/5	1/8	0/7					1/1						1/1	0/1	16/56 (29)	14/34 (41)
Pes planus	30/33	4/5	3/8													0/1	37/47 (79)	25/34 (73)
Pneumothorax															0/1	0/1	0/2 (0)	1/29 (3)
Premature teeth loss		1/5															1/5 (20)	
Proptosis																0/1	0/1 (0)	2/22 (9)
Protrusio acetabulae	7/20																7/20 (35)	
Retinal detachment																0/1	0/1 (0)	1/26 (4)
Retrognathia																0/1	0/1 (0)	6/29 (21)
Scoliosis	22/36		1/8	5/7			9/18			2/2					0/1	0/1	39/74 (53)	21/38 (55)
Severe allergy																0/1		3/23 (13)
Spondylysis/spondylilisthesis	10/26			1/7												0/1	11/34 (32)	8/27 (30)
Striae	17/32	2/5	0/8	1/7						1/2						1/1	22/55 (40)	5/28 (18)
Sudden death		1/7					15/50										16/57 (28)	
Tooth enamel defect																0/1	0/1 (0)	1/12 (8)
Translucent skin			2/8			0/1										1/1	3/10 (30)	7/30 (23)
Varices	18/31			1/7													19/38 (50)	
Velvety skin	18/29		0/8	1/7		0/1											19/45 (42)	
