# Supplementary material for: A mutation update on the LDS‐associated genes TGFB2/3 and SMAD2/3
Source: Hum Mutat. 2018 Mar 6;39(5):621–34. doi: 10.1002/humu.23407 (PMC5947146; doi:10.1002/humu.23407)
Supplement: Supplementary file 4 — Supporting Information Table S4 [file HUMU-39-621-s004.pdf]

Supplementary Table S4: Clinical features of all *TGFB3* patients reported so far.

**Clinical feature**	**Total (%)**
Hypertelorism	20/45 (45)
Downslant palpebral fissures	6/40 (15)
Retrognathia	10/20 (50)
Ectopia Lentis	0/11 (0)
Cataract	1/11 (9)
Exotropia	0/10 (0)
Myopia	6/15 (40)
Cleft Palate	7/13 (54)
Craniosynostosis	0/9 (0)
Blue sclerae	2/10 (20)
Malar Hypoplasia	3/10 (30)
Dolichocephaly	4/14 (29)
Retinal detachment	0/9 (0)
Glaucoma	0/8 (0)
Proptosis	1/10 (10)
Broad/bifid uvula	19/24 (79)
Dolichostenomelia	5/13 (39)
Joint hyperlaxity	21/26 (80)
Club foot	3/12 (25)
Flat feet	19/23 (83)
Osteoporosis	1/9 (11)
Pectus deformity	13/20 (65)
Cervical spine instability	1/10 (10)
Scoliosis	20/29 (69)
Joint dislocation	4/11 (36)
Arachnodactyly	18/45 (40)
Camptodactyly	3/12 (25)
Spondylolisthesis	2/10 (20)
Osteo-arthritis	5/11 (46)
Fractures	1/9 (11)
Thin, translucent skin	3/14 (21)
Striae	4/18 (22)
Delayed wound healing	2/16 (13)
Hernia	9/17 (53)
Dural ectasia	0/7 (0)
Easy bruising	8/15 (53)
Atrophic scarring	0/16 (0)
Artery dissection	0/9 (0)
Aortic dissection/rupture	5/16 (31)
Aortic surgery	3/13 (23)
Aortic root aneurysm	8/24 (33)
Ascending aortic aneurysm	4/17 (24)
Other aortic aneurysm	6/20 (30)
Arterial aneurysm	2/11 (18)
Aortic tortuosity	1/7 (14)
Arterial tortuosity	1/8 (13)
Mitral valve prolapse	6/15 (40)
Bicuspid aortic valve	1/10 (10)
Patent ductus arteriosus	0/9 (0)
Atrial septal defect	1/11 (9)
Pneumothorax	1/11 (9)
Tooth enamel defect	1/10 (10)
Eosinophilic esophagitis	0/8 (0)
Inflammatory bowel disease	1/9 (11)
Food allergy	1/10 (10)
Severe allergy	0/9 (0)
Nasal Polyps	0/8 (0)
