# Supplementary material for: A mutation update on the LDS‐associated genes TGFB2/3 and SMAD2/3
Source: Hum Mutat. 2018 Mar 6;39(5):621–34. doi: 10.1002/humu.23407 (PMC5947146; doi:10.1002/humu.23407)
Supplement: Supplementary file 5 — Supporting Information Table S5 [file HUMU-39-621-s005.pdf]

Supplementary Table S5: Clinical features of all *SMAD2* patients reported so far.

**Clinical feature**	**Total (%)**
Hypertelorism	1/6 (17)
Downslant palpebral fissures	2/2 (100)
Ectopia Lentis	0/2 (0)
Cataract	1/2 (50)
Exotropia	0/2 (0)
Myopia	0/2 (0)
High arched Palate	5/7 (71)
Cleft Palate	0/2 (0)
Blue sclerae	1/1 (100)
Malar Hypoplasia	1/1 (100)
Dolichocephaly	1/1 (100)
Broad/bifid uvula	1/7 (14)
Dolichostenomelia	1/3 (33)
Joint hyperlaxity	0/5 (0)
Club foot	0/1 (0)
Flat feet	3/5 (60)
Pectus deformity	3/8 (38)
Scoliosis	4/9 (44)
Arachnodactyly	3/6 (50)
Camptodactyly	0/4 (0)
Spondylolisthesis	0/1 (0)
Osteo-arthritis	3/3 (100)
Thin, translucent skin	0/5 (0)
Striae	3/7 (43)
Delayed wound healing	0/1 (0)
Hernia	4/5 (80)
Easy bruising	2/4 (50)
Atrophic scarring	0/3 (0)
Artery dissection	2/4 (50)
Aortic dissection/rupture	0/4 (0)
Aortic surgery	1/1 (100)
Aortic root aneurysm	5/6 (83)
Ascending aortic aneurysm	3/3 (100)
Other aortic aneurysm	1/2 (50)
Arterial aneurysm	1/3 (33)
Aortic tortuosity	0/4 (0)
Arterial tortuosity	1/4 (25)
Mitral valve prolapse	2/4 (50)
Bicuspid aortic valve	1/3 (33)
Severe allergy	0/4 (0)
